# Oscillatory Mechanism of α-Fe(N) ↔ γ’-Fe_4_N Phase Transformations during Nanocrystalline Iron Nitriding

**DOI:** 10.3390/ma15031006

**Published:** 2022-01-27

**Authors:** Walerian Arabczyk, Katarzyna Skulmowska, Rafał Pelka, Zofia Lendzion-Bieluń

**Affiliations:** Department of Inorganic Chemical Technology and Environment Engineering, Faculty of Chemical Technology and Engineering, West Pomeranian University of Technology in Szczecin, Piastów Ave. 42, 71-065 Szczecin, Poland; walerian.arabczyk@zut.edu.pl (W.A.); katarzyna.skulmowska@zut.edu.pl (K.S.); Zofia.Lendzion-Bielun@zut.edu.pl (Z.L.-B.)

**Keywords:** nanocrystalline iron, nitriding process, kinetics, oscillations, ammonia

## Abstract

The kinetics of nanocrystalline α-iron nitriding to γ’-iron nitride in an ammonia atmosphere was studied at 598–648 K and at atmospheric pressure. Oscillatory changes in nitriding reaction rates depending on nitrogen concentration in a solid sample were observed. This phenomenon was explained by a gradual change in the iron active surface coverage degree, with nitrogen resulting from a gradual change in the free enthalpy of nitrogen segregation. The α-Fe(N) nanocrystallites’ transformation into γ’-Fe_4_N went through six metastable FeN_x_ states. The continuous function proposed by Fowler and Guggenheim was modified to a stepwise variable function.

## 1. Introduction

Due to the practical applications of the iron and steel nitriding process, it was studied in the presence of coarse-crystalline iron and thin iron foils [1,2,3,4,5]. During these studies, Grabke, among others, observed that the surface chemical reaction is a rate-limiting step only in the initial phase of the process [3,4]. Later in the process, the diffusion of substrate through the product layer limits the nitriding rate.

In order to understand the nitriding mechanism, a simpler model system was used, in which nanocrystalline iron reacts with gaseous ammonia. In the case of nitriding of nanocrystalline iron, the effect of the diffusion of substances through the product layer on the process rate may be neglected due to the small size of nanoparticles and, thus, short (compared to coarse-crystalline materials) mass transport paths. In the process of nitriding of nanocrystalline iron there are two parallel reactions: the iron nitriding reaction, and catalytic decomposition of ammonia. The kinetics of the nanocrystalline iron nitriding process are presented in many other works [6,7,8,9,10]. Nitriding processes were conducted in the kinetic region at the nitriding potential of the gas phase, defined as P = p_NH3_/p_H2_^3/2^—much higher than the steady state potential (P^eq^)—at temperatures (T) above 673 K [7]. Smaller nanocrystallites of iron achieved the nitrogen critical concentration in their volume (X_N_^cri^) faster than larger ones [11]. Consequently, it was observed that nanocrystallites underwent the phase transformation according to their sizes, ranging from the smallest up to the largest. The phase transformation in a single iron nanocrystallite occurs throughout its volume. A model of reaction in the nanocrystalline materials–gas phase system in the adsorption area was developed [12], where it was stated that the dissociative adsorption rate of the gas phase on the nanocrystallite surface limited the rate of chemical reaction.

In the isothermal processes of nitriding of nanocrystalline iron, as well as reduction of nanocrystalline iron nitrides using ammonia–hydrogen gas mixtures at a determined nitriding potential, stationary states occurred [13,14]. A hysteresis was observed for the dependence of the nitriding degree on nitriding potential [13,14,15,16,17]. It was also found that the chemical potentials of nitrogen occurring in all three parts of the system—in the gaseous phase, on the iron nanocrystallite surface and in nanocrystallite volume — equaled one another in the stationary states, i.e., chemical equilibrium was established [16,18,19]. There was only a reaction of catalytic ammonia decomposition [20]. The minimum nitriding potentials required to initiate nitriding of nanocrystalline iron and reduction of nanocrystalline nitrides are the linear functions of nanocrystallite sizes [8,18,19,21]. Phase transformation of nanocrystallites in close–to-equilibrium states takes place in the entire volume of nanocrystallites according to their sizes, starting from the largest down to the smallest [16,22]. Based on theoretical calculations concerning thermodynamic parameters, it was found that such an order of nanocrystallite transformation occurred in the nanocrystalline iron nitriding process as well as in the reduction of nanocrystalline iron nitrides [18,19]. In the nanocrystalline iron nitriding process with ammonia–hydrogen mixtures, two solid phases occur simultaneously at the determined nitriding potential, and three solid phases in the wide ranges of nitriding potential occur simultaneously in the process of reducing the nanocrystalline ε iron nitrides [15,23]. The Lehrer diagram does not explain the above-mentioned phenomena taking place in a system of nanocrystalline iron, ammonia, and hydrogen [24]. In order to describe these phenomena, an additional intensive parameter—the size of nanocrystallites—was introduced into the extended Gibbs phase rule [19].

The oscillatory kinetics of heterogeneous reactions have already been observed for systems involving phenomena on the surface of the solid phase [25,26,27,28,29,30,31,32,33,34,35,36,37,38,39,40,41]. It has been found that oscillatory states are caused by periodic variations in the degree of catalysts’ surface coverage. Saraev et al. [42] observed oscillatory phenomena in solid-phase–gas-phase reaction systems with phase transformation of a solid substrate in the surface layer (a range of several dozens of atomic layers). As a result of detailed kinetic studies of the nitride reduction process, we observed oscillatory changes in the rate of the reduction reaction of iron γ’-Fe_4_N iron nitride to α-iron [43]. The observed oscillatory phenomenon has been explained by a gradual variation in the free enthalpy of nitrogen segregation.

It can be concluded that the above phenomena are not observed in coarse-crystalline materials with a negligible specific surface, where the diffusion rate of the substrate through the product layer limits the rate of chemical reaction. In the case of these substances, the influence of surface reactions is not taken into account precisely because of the low value of the specific surface area. In the case of nanocrystalline substances, the share of surface and surface reactions is significant. Then, the surface energy plays an important role, and surface phenomena contribute to the free enthalpy of the whole chemical process. The transformation depends on the balance of both surface energy and that related to the volume of nanocrystallites. Thus, the aim of this work was to explain the phenomenon of the periodicity of chemical reaction rates in the case of nanocrystalline materials, and nanocrystalline iron nitriding with ammonia was used as an example of the system under study.

## 2. Materials and Methods

An industrial iron catalyst for ammonia synthesis—KM1R (pre-reduced form containing nanocrystalline iron doped with hardly reducible oxides of aluminum (3.3 wt.%), calcium (2.8 wt.%), and potassium (0.65 wt.%))—was used in the study. The average size of iron nanocrystallites prior to the nitriding process, as determined by X-ray diffraction (Rietveld method), was 45 nm. The specific surface area of the catalyst, as determined by the thermal desorption method, was 12 m^2^/g [44]. Nitriding reaction rate measurements were conducted in a differential tubular reactor with both a thermogravimetric measuring system and a hydrogen analyzer. A solid sample of 1 g was placed in the form of a single layer of grains (1.0–1.2 mm) in a platinum reactor basket suspended on the arm of a thermobalance. Ammonia (99.98%) and hydrogen (99.999%) were applied. The description of the installation is presented elsewhere [2]. During the exchange of hydrogen (present in the reactor) for ammonia, the composition of the gas phase in the two-component system was changing. Model calculations of changes in the composition of the gas phase in the reactor reaction volume were performed, taking into account the simplified differential mass balance equation of the reactor with perfect mixing:

(1) $$-\frac{dX}{dt}=\frac{F_{0}\left(X_{i}-X\right)}{U}$$

where *X_i_* is the concentration (mol/mol) (feed stream), *F*_0_ is the molar flow rate (mol/s), *U* is the total number of moles in the reactor volume, in which the reaction takes place, and *t* is the time (s). 

The value of *F*_0_ was 0.00015 moL/s. By modeling Equation (1) with the experimental results, a value of *U* = 0.008 moL was determined, corresponding to a reaction volume of 180 cm^3^.

**Figure 1:**
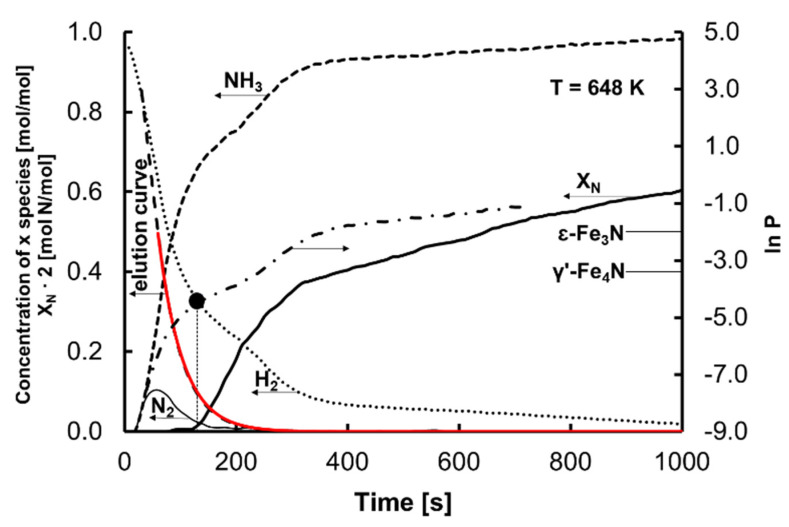
Dependence of the concentration of nitrogen in the nanocrystalline iron sample, the concentrations of hydrogen, ammonia, and nitrogen, and the nitriding potential on time for the nanocrystalline iron nitriding process (100% ammonia at the reactor inlet) at 648 K. The continuous red line shows the model results of the elution curve.

Figure 1 contains the dependence of hydrogen concentration on time (the so-called “elution curve”) and a comparison of the results calculated with the experimental data. Based on the compatibility of the model and experimental results for hydrogen concentration measurements performed over and under the catalyst sample basket, it was found that under the experimental conditions in the reaction volume there was a state close to an ideal gas-phase mixing. The iron catalyst was subjected to a polythermal hydrogen reduction process, with temperature increasing from room temperature to 773 K at a rate of 0.24 K/s. The sample was reduced at 773 K until a constant mass was obtained. Selected measurement results concerning nanocrystalline iron nitriding with ammonia carried out isothermally at 648, 623, and 598 K are presented in this publication. During the nitriding process, ammonia was dosed into the reactor, and a gradual change in the gas-phase composition took place from hydrogen—present in the reactor after the catalyst reduction process—to ammonia. The nitriding process was conducted at a variable nitriding potential up to the moment when a stationary state was established, during which the measured hydrogen concentration in the reactor was constant and the rate of the nitriding reaction was zero. The composition of samples of nitrided nanocrystalline iron depends on the nitriding potential and temperature [24,45]. Under these conditions, nanocrystallites undergo phase transformation in the order from the smallest to the largest.

## 3. Results

Figure 1 shows an example of the dependence of the nitrogen concentration in the nanocrystalline iron sample, the concentrations of the individual components in the gaseous phase, and the change in nitriding potential on time for the nanocrystalline iron nitriding process at 648 K. Based on the results of thermogravimetric measurements and determination of hydrogen concentration in the gas phase, taking into account the mass balance of the reactor with perfect mixing, ammonia and nitrogen concentrations and nitriding potential were calculated at each process step [7].

Based on the computed composition of the gas phase, the minimal nitriding potential (*P*_0_), at which the phase transformation in the nitriding process of nanocrystalline iron begins, was determined. For each temperature in the nanocrystalline iron nitriding process, the smallest nanocrystallite reacts at a different nitriding potential. Based on the investigations of nanocrystalline iron nitriding processes in stationary states for the dependence of logarithms of nitriding potential on reciprocal temperature, the minimum nitriding potential of the smallest nanocrystallite for the temperature range 573–673 K can be described by the following equation [10,13,15,16,17,23]:

(2) $$lnP_{0}=18.3+8.91\frac{1}{T}$$

In the nitriding process at a potential lower than the minimum nitriding potential at which the γ’-Fe_4_N phase is produced, samples of iron contained a small amount of nitrogen—X_N_ = 0.004 mol N/mol. At this stage of the nitriding process, an increase in the sample mass took place due to the adsorption of ammonia on the nanocrystalline iron surface and the formation of a solution of nitrogen in nanocrystalline iron, referred to as α-Fe(N). Based on Equation (2), the minimum nitriding potential in the nitriding process of nanocrystalline iron at different reaction temperatures was calculated. For the dependence of hydrogen concentration on time (Figure 1), the value at which the hydrogen concentration corresponding to the nitriding potential (*P*_0_) was reached is marked with a black point.

## 4. Discussion

**Figure 2:**
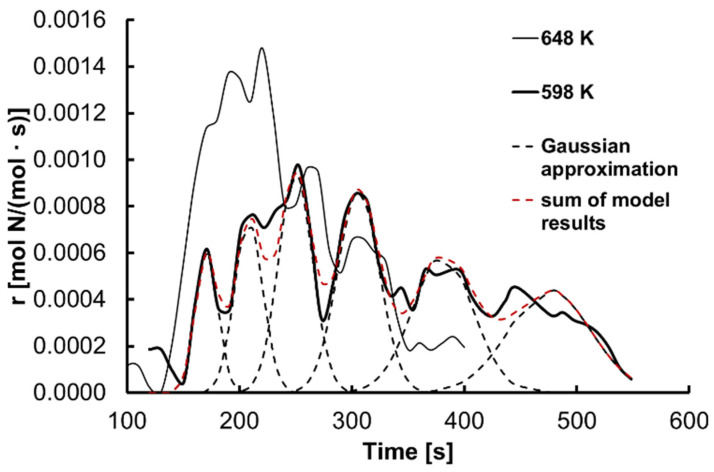
Dependence of the nitriding rate of nanocrystalline iron at 598 and 648 K on time. The red dashed line indicates the total reaction rate resulting from the proposed model.

Based on the dependence of nitrogen concentration in the sample on the time of the nanocrystalline iron nitriding, the rates of this process were calculated as shown by time for temperatures of 648 and 598 K in Figure 2. For the dependence of the nanocrystalline iron nitriding rate on the time of the process, the occurrence of oscillatory reaction rate changes was observed. These oscillations were observed throughout the temperature range under investigation.

**Figure 3:**
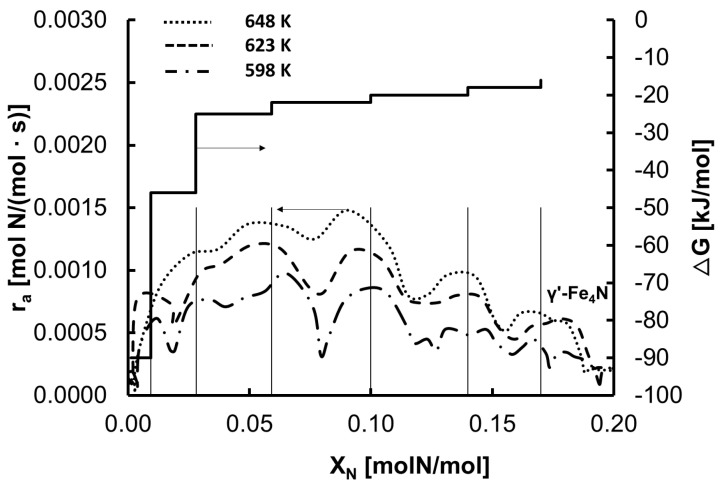
Dependence of the nanocrystalline iron nitriding rate and free enthalpy of nitrogen segregation to the nanocrystalline iron surface on the concentration of nitrogen in the solid sample.

Making use of both the results presented in Figure 1 and the nitriding reaction rate shown in Figure 2, the dependence of the rates of the processes studied on nitrogen concentration in the sample were determined (Figure 3). The observed oscillations of the nitriding reaction rate depended on the nitrogen concentration in the iron.

The vertical continuous lines represent nitrogen concentrations at which the maximum nanocrystalline iron nitriding rate was observed. It was found that the six observed maxima in the nitriding process occurred at constant values of nitrogen concentration in iron, taking into account the measurement accuracy, at different temperatures.

Nanocrystalline iron nitriding processes were conducted under isothermal conditions with a variable nitriding potential. The transformation of the α-Fe(N) phase to γ’-Fe_4_N starts with the minimum nitriding potential necessary to initiate the transformation of the smallest α-Fe nanocrystallite into the γ’ phase. The average nitriding rate for the entire set of nanocrystallites is expressed by the following equation [46,47,48,49]:

(3) $$r=k\left(S_{sp}\;-\;S_{pr}\right)\left(P-P_{0}\right)f\left(\alpha \right)$$

where *k* is the constant of the nitriding reaction rate, *S_sp_* is the specific surface area, *S_pr_* is the surface occupied by promoters, *P* is the current nitriding potential, and *f*(*α*) is the conversion degree function.

For a single nanocrystallite of a medium size, the nanocrystalline iron nitriding process with ammonia was modeled numerically. The nitriding rate of this nanocrystallite is expressed as follows:

(4) $$r_{i}=k\;{\left(S_{sp}\;-\;S_{pr}\right)}_{i}\left(1-\theta_{i}\right)f{\left(P\right)}_{i}$$

where (*S_sp_* − *S_pr_*) is the specific active surface area of nanocrystallite, *θ* is the active surface coverage degree, and the subscript *i* represents the *i*-th nanocrystallite.

The above equation is valid for the range of 0 < X_N_ < X_N_^cri^.

In nanomaterials, the diffusion path of nitrogen in the volume of nanocrystallites is very short, so the concentration in the volume is close to the equilibrium values. There is little difference between the potential of nitrogen adsorbed on the surface of the solid (μ_s_) and that dissolved in the solid’s volume (μ_b_):

μ_g_ = μ_s_ ≈ μ_b_ (5)

In this case, the nitriding process rate is not limited by the adsorption process rate. Chemical equilibrium between the substance adsorbed on the nanocrystallite surface and that dissolved in its volume can be described by McLean–Langmuir equation [50]:

(6) $$\frac{\mathrm{KP}}{1-\mathrm{KP}}=\frac{\gamma X_{b}}{1-\gamma X_{b}}\exp\left(\frac{-\Delta G}{\mathrm{RT}}\right)\;$$

where Δ*G* is the free enthalpy of segregation of a substance, R is the gas constant, *γ* is the activity coefficient, and *X_b_* is the nitrogen molar concentration.

Fowler and Guggenheim proposed [51] that the dependence of the free enthalpy of segregation (Δ*G*(*X_b_*)) on the concentration of substance (*X_b_*) in the nanocrystallite is linear, and is valid for one phase (stable according to the Fe–N thermodynamic system):

(7) $$\Delta G\left(X_{b}\right)=\Delta G_{0}+\alpha X_{b}$$

where *α* is the proportionality coefficient.

**Figure 4:**
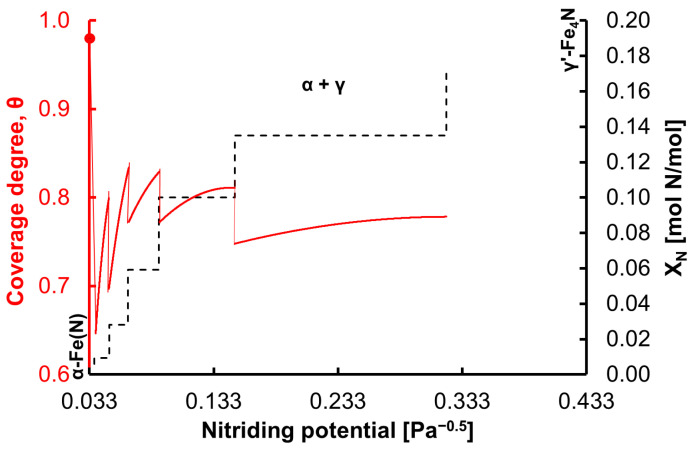
Dependence of coverage of the iron surface with nitrogen and concentration of nitrogen in the iron nanocrystallite volume on the nitriding potential.

In the state of chemical equilibrium, when the chemical potential of nitrogen is equal to the absolute value of the potential change in the deformed crystal lattice of nanocrystalline iron, the saturated solution of α-Fe(N)—which is in a state of chemical equilibrium—is transformed to an unsaturated solution of nitrogen in the γ-iron crystallographic phase—which, in turn, is in a state of transition—and further to γ’-Fe_4_N nitride, which is again in the chemical equilibrium state [18,19]. The step change in the nanocrystalline iron nitriding rate (Figure 2 and Figure 3) can be explained by the gradual change in the degree of coverage of the iron surface with nitrogen (Figure 4), caused by the gradual change in the free enthalpy of nitrogen segregation (Figure 3). At each step change, a metastable state of FeN_x_ is formed. Therefore, the continuous function (Equation (6)) was modified to a stepwise variable function. Transformation of a single α-Fe(N) nanocrystallite into γ’-Fe_4_N occurs via the formation of six metastable FeN_x_ states. In the first stage of the nitriding process, a solution of nitrogen in nanocrystalline iron—α-Fe(N)—is obtained, for which the free enthalpy of segregation equals –90 kJ/moL [52]. From the stable phase of α-Fe(N), after exceeding a critical concentration of ca. X_N_ = 0.004 mol N/mol, a metastable phase is formed, where maximum coverage of the surface with nitrogen is reached at a critical X_N_ value for the metastable phase after surface coverage is increased by an increase in the concentration of nitrogen in the nanocrystallite volume. Figure 4 shows the dependence of the coverage of the iron surface with nitrogen and the concentration of nitrogen in volume on the nitriding potential. The value of the minimum nitriding potential at which the phase transformation in the nitriding reaction starts from nanocrystalline iron to iron nitrides is taken from Figure 1. With the minimum nitriding potential *P*_0_ = 0.034 Pa^−0.5^, the degree of surface coverage of a single iron nanocrystallite—α-Fe(N)—with nitrogen is *θ* ≈ 0.999 (for Δ*G* = −90 kJ/mol). Changes in the surface coverage during the single iron nanocrystallite nitriding process for subsequent metastable phase transformations at 598 K were calculated based on Equations (4) and (6). Based on Equation (7), values of the free enthalpy of segregation were calculated, and the results are shown in Figure 3 in relation to the nitrogen concentration in iron.

The measured rate of the reaction is the sum of the process rates for a given set of nanocrystallites in the sample, as follows:

(8) $$r_{}=\overset{i=n}{\underset{i=1}{\sum }}r_{i}^{}$$

According to Equation (4), the nitriding reaction rate is a function of the nanocrystallites’ specific active surface area distribution [46,47,48,49]. The maximum rate of formation of individual metastable phases corresponds to the maximum of the specific active surface area distribution of nanocrystallites. Assuming that the crystallites’ specific active surface area distribution is normal (Gaussian), Figure 2 shows the dependence of the nanocrystalline iron nitriding rate on time, on which the subsequent transformations of the metastable phases are marked. For such a distribution, the maximum corresponds to the average specific active surface area of the nanocrystallites. The critical nitrogen concentrations in iron nanocrystallites corresponding to the maximum rate of formation of the six metastable phases are 0.01, 0.03, 0.06, 0.10, 0.14, and 0.17 mol N/mol, respectively. Determined values are indicated by vertical lines in Figure 3.

## 5. Conclusions

In the nitriding process of nanocrystalline iron in an ammonia atmosphere, six oscillating changes in the nitriding rate of nanocrystalline iron were observed. The oscillations depended on the concentration of nitrogen in the solid sample. The oscillations occurring during the nitriding of nanocrystalline iron are related to the specific active surface area distribution of nanocrystallites, as well as to the formation of metastable nitrides. The observed transition between stable phases is caused by step changes in the free enthalpy of segregation, resulting in phase changes.
